# Self-healing and shape-memory polymers based on cellulose acetate matrix

**DOI:** 10.1080/14686996.2024.2320082

**Published:** 2024-02-19

**Authors:** Han Jia, Keiya Jimbo, Hirogi Yokochi, Hideyuki Otsuka, Tsuyoshi Michinobu

**Affiliations:** a Department of Materials Science and Engineering, Tokyo Institute of Technology, Meguroku, Tokyo, Japan; b Department of Chemical Science and Engineering, Tokyo Institute of Technology, Meguroku, Tokyo, Japan

**Keywords:** Self-healing, shape memory, biodegradable, cellulose acetate, ureidopyrimidinone

## Abstract

The creation of self-healing polymers with superior strength and stretchability from biodegradable materials is attracting increasing attention. In this study, we synthesized new biomass-derived cellulose acetate (CA) derivatives by ring-opening graft polymerization of δ-valerolactone followed by the introduction of ureidopyrimidinone (Upy) groups in the polymer side chains. Due to the semicrystalline aliphatic characteristics of the side chain poly(δ-valerolactone) (PVL) and quadruple hydrogen bonds formed by the Upy groups, the stretchability of the resulting polymers was significantly enhanced. Moreover, the shape memory ability and self-healing property (58.3% of self-healing efficiency) were successfully imparted to the polymer. This study demonstrates the great significance of using biomass sources to create self-healing polymers.

## IMPACT STATEMENT

This paper describes the first successful demonstration of self-healing polymers with superior strength and stretchability from a biodegradable material, cellulose acetate (CA). We initially introduced the ureidopyrimidinone (Upy) groups in the side chains of CA. However, the resulting polymer was not soluble and processable. In order to solve this issue, a new strategy based on the ring-opening graft polymerization of δ-valerolactone followed by the introduction of ureidopyrimidinone (Upy) groups was adopted. Due to the semicrystalline aliphatic characteristics of the side chain poly(δ-valerolactone) (PVL), the resulting polymers were soluble and processable. In addition, the quadruple hydrogen bonds formed by the Upy groups enhanced the stretchability of the resulting polymers. Moreover, the shape memory ability and self-healing property were successfully achieved due to the presence of PVL and Upy. The developed new strategy can be applied to a variety of polymers including biomass-based polymers and materials.

## GRAPHICAL ABSTRACT

**Figure:**
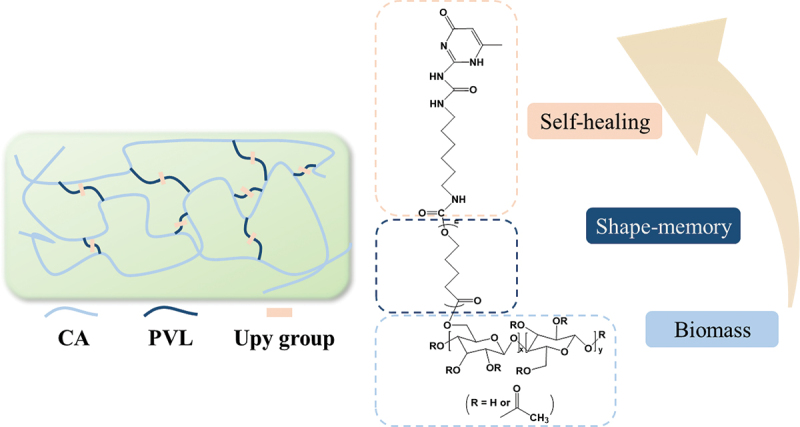

## 1. Introduction

Self-healing polymers are defined as polymers that have the ability to restore physical properties and repair damage with minimum intervention or with specific stimuli [1–4]. Due to their outstanding mechanical properties and long lifetime endowed by self-healing characteristics, self-healing polymers have been intensively investigated for applications in adhesives [5–7], electronics [8–14], thermoconductive skins [15], biomedical [16–19], soft actuators [20,21], etc.

In the last decade, more and more studies have found that self-healing polymers can be effectively synthesized by introducing dynamic bonds, which are classified as dynamic covalent and noncovalent bonds [22–24]. Dynamic covalent bonds include dynamic transesterification [25–27], dynamic disulfide bonds [28–32], and Ru-Se coordination [33]. Moreover, it is worth mentioning that Diels-Alder (D-A) adducts have also been widely used in dynamic polymers to endow the polymers with self-healing ability via the reversible Diels-Alder and retro-Diels-Alder reaction at elevated temperatures [34]. Compared to dynamic covalent bonds, noncovalent bonds have lower binding energy and can respond more quickly to external stimuli and induce repair. Therefore, self-healing polymers based on dynamic noncovalent bonds are attracting attention. Common dynamic noncovalent bonds include coordination bonding [35,36], π–π stacking interactions [37,38], and hydrogen bonding interactions [39,40]. Although polymers based on dynamic noncovalent bonds exhibit excellent self-healing efficiency, their relatively weak mechanical properties due to the mismatch between reversibility and stability cannot be ignored. Fortunately, the ureidopyrimidinone (Upy) groups were found to effectively increase the stiffness and toughness of polymers by forming quadruple hydrogen bonds, which can impart self-healing ability [41]. The effects of Upy groups on the formation of physical crosslinking networks and the physical properties of polymers have been intensively investigated during the past decades [42–44]. For instance, Bao et al. reported a supramolecular polymer synthesized by soft polymeric chains (polytetramethylene glycol and tetraethylene glycol) and Upy cross-linkers [45]. The resulting polymer showed extremely high stretchability (up to 17,000% strain), toughness, and self-healing properties. In addition, the good interfacial adhesion to the gold thin film allows the gold thin film electrode deposited on this polymer to retain its conductivity and fracture/notch insensitivity. By modifying TEMPO-oxidized cellulose nanofibers with Upy groups and introducing disulfide bonds in the polyurethane main chain, Yang and co-workers fabricated a novel polyurethane/nanocellulose elastomer with outstanding self-healing, self-reinforcing, and toughening performances [8]. Due to the dual dynamic cross-linking networks formed in the composite, the elastomer was fully self-healed within 4.0 h at 50°C.

**Scheme 1.**
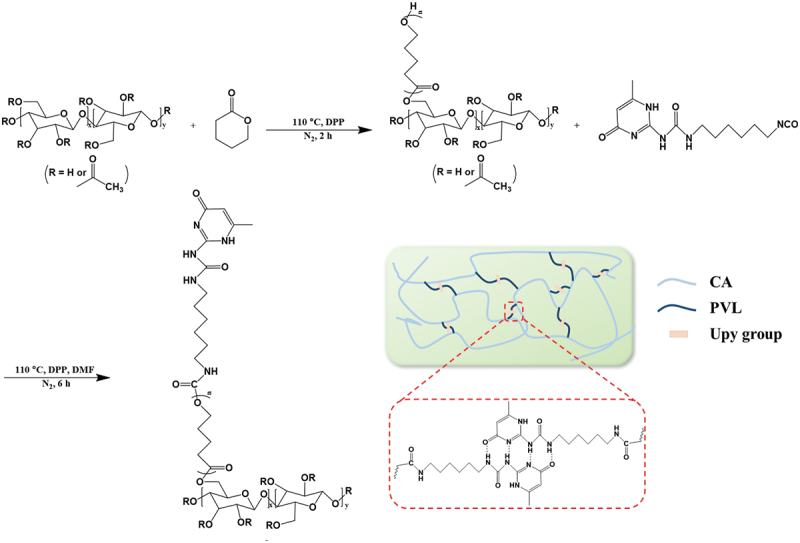
Schematic diagram of the synthesis procedure for CA-g-PVL-Upy and the quadruple hydrogen bond interactions of the Upy groups in the polymer.

In general, petroleum-derived non-degradable crude materials are widely used in the production of self-healing polymeric chains [46]. The development of self-healing polymeric materials with biodegradable and environmentally friendly materials is of great significance [47–49]. Cellulose acetate (CA), a cellulose derivative, is a relatively inexpensive biobased polymer that exhibits good mechanical properties, highly chemical modification possibility, and biodegradability [50,51]. Therefore, CA was used as the polymer main chain in this research. As shown in Scheme 1, biodegradable poly(δ-valerolactone) (PVL) was grafted into CA by ring-opening graft polymerization to improve the flexibility and stretchability of CA [52]. The Upy groups were then introduced into the PVL chain terminals. Based on the crystalline feature of PVL and the quadruple hydrogen bonds formed by Upy, the resultant polymers were simultaneously endowed with self-healing and shape memory capability.

## 2. Materials and methods

### 2.1. Materials

Cellulose acetate (CA, *M*_n,GPC_ = 84.5 × 10^3^ g mol^−1^, polydispersity index PDI = 3.00) with the degree of substitution (*DS*) of 2.4 was supplied from Nippon Paper Industries Co., Ltd. (Tokyo, Japan). Diphenyl phosphate (DPP), δ-valerolactone (VL), 2-amino-4-hydroxy-6-methylpyrimidine, and hexamethylene diisocyanate (HDI) were purchased from Tokyo Chemical Industry Co., Ltd. (Tokyo, Japan). *N,N*-Dimethylformamide (DMF) was purchased from Kanto Chemical Co., Inc. (Tokyo, Japan).

### 2.2. Synthesis of 2-(6-isocyanatohexylaminocarbonylamino)-6-methyl-4[1 H]-pyrimidinone (Upy-NCO)

2-(6-Isocyanatohexylaminocarbonylamino)-6-methyl-4[1 H]-pyrimidinone (Upy-NCO) was synthesized according to the reported method [53,54]. A solution of 2-amino-4-hydroxy-6-methylpyrimidine (175.2 mg, 1.4 mmol) in HDI (1.6 g, 9.5 mmol) was heated at 100°C for 16 h under N_2_ protection. Pentane was then added to the mixture and the resulting precipitate was filtered and washed with pentane repeatedly. The resulting white powder was dried in a vacuum oven at 60°C for 12 h. The chemical structure was confirmed by proton nuclear magnetic resonance (^1^H-NMR) (Figure S1).

### 2.3. Ring-opening graft polymerization of VL onto CA

The ring-opening graft polymerization of VL was carried out with DPP as the catalyst. A typical procedure is as follows: CA (285 mg, 1.08 mmol in anhydroglucose residue) and VL (4.0 g, 40 mmol) were added into the flask and the system was heated at 110°C with stirring under a N_2_ atmosphere. After 30 min, the mixture became transparent liquid, and DPP (20 mg, 0.08 mmol) was then added. Continuous stirring was conducted for another 120 min, which is defined as the reaction time of the graft polymerization. After the graft polymerization was completed, 10 mL of chloroform was added into the reaction mixture. The resulting homogeneous solution was dropwise poured into a vigorously stirred, large excess of methanol/toluene mixture (7:3 in volume). The graft polymer product (CA-g-PVL) obtained as a precipitate was filtered and dried at 60°C in vacuum for 12 h. *M*_n,GPC_ = 214 × 10^3^ g mol^−1^, PDI = 1.98. ^1^H NMR (CDCl_3_): *δ* = 1.53–1.68 (4 H, m), 1.83–2.13 (9 H, m), 2.33–2.36 (2 H, m), 3.65–3.68 (2 H, m), 4.07–4.10 (2 H, m).

The ^1^H NMR data were used for the determination of the molar substitution (*MS*) and the degree of oxyalkanoyl substitution (*oxyalkanoyl DS*), defined as the average number of oxyalkanoyl units introduced and the number of hydroxyls substituted for oxyalkanoyls, respectively, per anhydroglucose residue of CA. In the ^1^H NMR spectrum of CA-g-PVL (Figure S2), the resonance peak area derived from the methyl protons of acetyl groups was designated as *A*, the area of the resonance signals from the protons of oxyalkanoyls was designated as *B*, and the area from the terminal protons of oxyalkanoyls was designated as *C* (*A:B:C* = 1:6.19:0.25). The *MS*, oxyalkanoyl *DS*, and the degree of polymerization of the side chain (${\bar{\mathrm{DP}}}_{s}$) can be calculated by the following equations:

(1) $$\mathrm{MS}=\frac{\left(\mathrm{acetyl}\;\;\mathrm{DS}\right)\left(\frac{B+C}{n}\right)}{\frac{A}{3}}$$

(2) $$\mathrm{oxyalkanoyl}\;\;\mathrm{DS}=\frac{\left(\mathrm{acetyl}\;\;\mathrm{DS}\right)\left(\frac{C}{n}\right)}{\frac{A}{3}}$$

(3) $${\bar{\mathrm{DP}}}_{s}=\frac{\mathrm{MS}}{\mathrm{oxyalkanoyl}\;\;\mathrm{DS}}$$

where *n* is equal to 2, denoting the number of protons of the species selected for the calculation of oxyalkanoyl *MS* and *DS* [55]. The obtained *MS*, *oxyalkanoyl DS*, and ${\bar{\mathrm{DP}}}_{s}$ were 23.18, 0.9, and 25.8, respectively. The average molecular weight of CA-g-PVL unit was 1822.2 g mol^−1^.

### 2.4. Preparation of CA-g-PVL-Upy by modification of CA-g-PVL with Upy-NCO

CA-g-PVL-Upy was synthesized in DMF solution. The dried CA-g-PVL (1.0 g, 0.33 mmol in hydroxyl groups) and Upy-CNO (0.096 g, 0.33 mmol) were dissolved in 10 mL of DMF with stirring at 110°C under N_2_ atmosphere. After 30 min, DPP (20 mg, 0.080 mmol) was added. Continuous stirring was conducted for another 6 h. By drying the resulting solution at 110°C in vacuo for 24 h, a CA-g-PVL-Upy film was obtained. *M*_n,GPC_ = 259 × 10^3^ g mol^−1^, PDI = 2.07. ^1^H NMR (CDCl_3_): *δ* = 1.60–1.68 (4 H, m), 1.83–1.95 (9 H, m), 2.05 (3 H, s), 2.33–2.36 (2 H, m), 3.15–3.24 (2 H, m), 4.07–4.10 (2 H, m), 4.80–4.93 (1 H, m), 5.86 (1 H, s), 10.13 (1 H, s), 11.87 (1 H, s), 13.14 (1 H, s).

### 2.5. Characterization

The chemical structures were analyzed by Fourier transform infrared (FTIR) spectroscopy (FT/IR-4200, JASCO, Tokyo, Japan) from 4000 to 500 cm^−1^ at 20°C. ^1^H- NMR spectra were measured on a 400YH NMR spectrometer (JEOL, Tokyo, Japan). Thermogravimetric analysis (TGA, Thermoplus TG8120, Tokyo, Japan) was carried out from 20°C to 500°C under flowing nitrogen at the scan rate of 10°C min^−1^. The differential scanning calorimetry (DSC, DSC8230, Thermo Plus EVO, Tokyo, Japan) measurements were conducted at the scan rate of 10°C min^−1^ from −80 to 150°C under nitrogen flow. Gel-permeation chromatography (GPC) was performed at 40°C using a JASCO HSS-1500 system (Tokyo, Japan) with a refractive index (RI) detector. *N,N*-Dimethylformamide (DMF) with lithium bromide (5 mM) was used as the eluent at a flow rate of 0.6 mL min^−1^. Polystyrene standards (number average molecular weight (*M*_n_): 4430–3 242 000 g mol^−1^; polydispersity index (*M*_w_/*M*_n_): 1.03–1.08) were used to calibrate the GPC system. The strain-stress curves of CA, CA-g-PVL, and CA-g-PVL-Upy were measured using a testing machine (Toyoseiki Strograph-VES5D, Tokyo, Japan) at the rate of 10 mm min^−1^.

## 3. Results and discussion

### 3.1. Structural characterization

**Figure 1.**
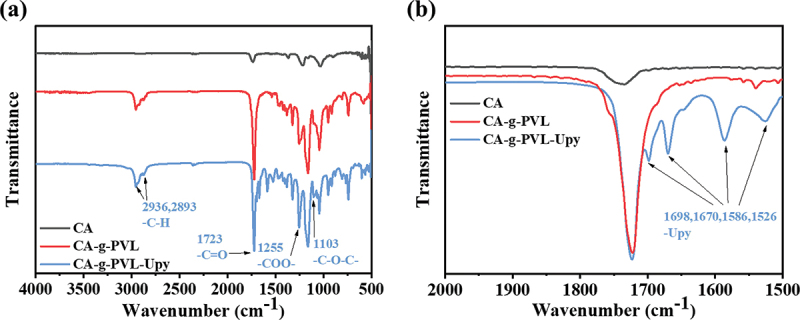
(a) FTIR spectra of CA, CA-g-PVL, and CA-g-PVL-Upy and (b) the enlarged spectra in the wavenumber range from 1500 to 2000 cm^−1^.

The chemical structures of the resulting polymers were characterized by ^1^H NMR (Figure S1–3) and FTIR spectra. As shown in Figure 1(a), after ring-opening graft polymerization of VL onto CA, two broad peaks appeared at 2936 and 2893 cm^−1^ assigned to the C–H stretching bands of –CH_2_– and –CH_3_ in PVL, respectively. Additionally, the peaks at 1723, 1255, and 1103 cm^−1^, which are assigned to the –C=O, –COO–, and –C–O–C– groups, respectively, were significantly enhanced due to the grafting of PVL. As displayed in Figure 1(b), the peaks at 1670 and 1698 cm^−1^ in CA-g-PVL-Upy were attributed to the carbonyl stretching vibration of –C=O in urea and pyrimidinone, respectively. The peaks at 1586 and 1526 cm^−1^ corresponding to the –C=C– and aromatic ring vibrations of the Upy also suggested that the Upy group was successfully introduced into the PVL chains [56]. Moreover, the absence of the -NCO peak at 2274 cm^−1^ confirmed that all the –NCO groups fully reacted with the –OH in CA-g-PVL. Therefore, CA-g-PVL and CA-g-PVL-Upy were successfully synthesized.

Due to the existence of hydroxyl groups in CA chains, Upy-NCO directly reacts with CA. To investigate the effect of the PVL graft chains on the resultant products, we synthesized CA-Upy as a control sample according to the synthesis procedure shown in Figure S4(a). The chemical structure of the obtained CA-Upy was characterized by^1^H NMR (Figure S5). It is noted that as CA-Upy precipitated during the reaction process, this polymer has a solubility issue. Therefore, CA-Upy did not form a film by solvent-casting as CA-g-PVL-Upy did (Figure S4(b,c)), due to the strong intermolecular interaction and the lack of flexible graft chains. The following tests were thus conducted on CA, CA-g-PVL, and CA-g-PVL-Upy.

The molecular weights of the obtained polymers were measured by GPC. The original CA showed a *M*_n_ of 84.5 × 10^3^ g mol^−1^ and large molecular weight distribution (PDC = 3.00), while CA-g-PVL displayed a much higher *M*_n_ (214 × 10^3^ g mol^−1^) and narrower molecular weight distribution (PDI = 1.98) (Figure S6). This result suggested the successful formation of graft polymers by the controlled ring-opening polymerization of VL. It is noticeable that a small amount of byproduct (*M*_n_ = 16.1 × 10^3^ g mol^−1^, PDI = 1.40) existed, which is attributed to the homopolymers of VL resulting from the polymerization induced by a trace amount of water in the reaction and/or small molecular weight CA chains [55]. With the further introduction of Upy groups, *M*_n_ of CA-g-PVL-Upy increased to 259 × 10^3^ g mol^−1^. However, *M*_n_ and PDI of the byproduct did not significantly change.

### 3.2. Thermal properties

**Figure 2.**
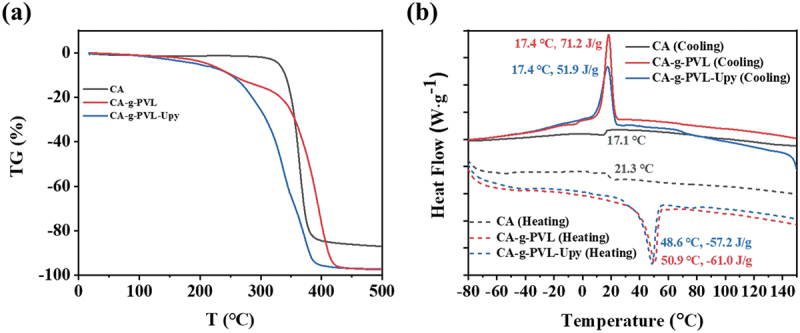
(a) TGA and (b) DSC curves of CA, CA-g-PVL, and CA-g-PVL-Upy measured at the scanning rate of 10°C min^−1^ under flowing nitrogen.

Thermal stability is a key factor related to the self-healing performance of self-healable polymers. Hence, the thermal properties of CA, CA-g-PVL, and CA-g-PVL-Upy were studied by TGA and DSC. As summarized in Figure 2(a) and Table 1, CA showed outstanding thermal stability with a high decomposition temperature (T_d_ = 329°C). It should be noted that T_d_ of CA-g-PVL significantly decreased to 213°C due to the introduction of flexible PVL graft chains. Compared with CA-g-PVL, CA-g-PVL-Upy showed a slightly lower T_d_ (196°C), which is attributed to the low T_d_ of the Upy-NCO unit (176°C) as shown in Figure S7. Although the introduction of Upy reduced the T_d_ of the polymer, the thermal stability of the final target polymer was sufficiently high considering the operating temperature of the quadruple hydrogen bonds of the Upy groups [8,53,57]. As a result, CA-g-PVL-Upy was self-healing upon thermal stimulation (vide infra).

**Table 1.** The decomposition temperatures (T_d_) estimated from TGA curves.

Sample	CA	CA-g-PVL	CA-g-PVL-Upy
T_d_ (℃) (TG = 5%)	329	213	196

The thermal transition behavior of the resultant polymers was investigated by DSC. As displayed in Figure 2(b), CA showed clear glass transition temperatures T_g_ at 17.1 and 21.3°C in the cooling and heating process, respectively. Consistent with the excellent crystallization ability of PVL, an obvious crystallization peak (17.4°C, 71.2 J g^−1^) and a melting peak (50.9°C, −61.0 J g^−1^) appeared in CA-g-PVL. Compared with CA-g-PVL, no significant differences in crystallization temperature, melting temperature, and the corresponding enthalpy were observed for CA-g-PVL-Upy. This result suggested that the introduction of Upy groups did not significantly change the crystallization of PVL. Additionally, the reduced melting temperature of CA-g-PVL-Upy indicates a possible shape memory capability based on the melting-recrystallization of the PVL chains.

### 3.3. Mechanical properties

**Figure 3.**
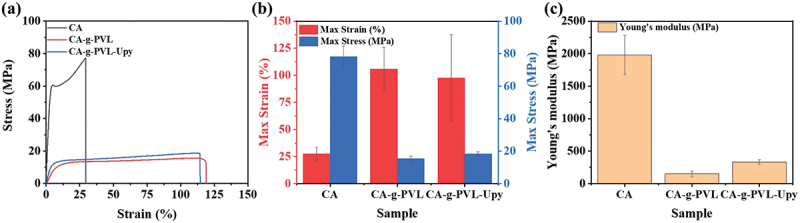
(a) Strain-stress curves of CA, CA-g-PVL, and CA-g-PVL-Upy. (b) The maximum strain and maximum stress values obtained from the strain-stress curves. (c) Young’s moduli estimated from the strain-stress curves.

Since the mechanical strength of polymers is an important factor in durability, the strain-stress curves of all samples were recorded (Figure 3(a) and Figure S8). All the test samples were cut into dumbbell shapes of the same size (length × width × thickness = 12 mm × 2 mm × 0.19 mm) before measurement. As shown in Figure 3(b,c), the pristine CA film had excellent high maximum stress (78.20 MPa), low maximum strain (27.40%), and high Young’s modulus (1980.25 MPa) due to the strong intermolecular forces between the CA chains [50,51]. The grafting of flexible PVL chains significantly increased the maximum strain of CA-g-PVL to 105.87%, and significantly decreased the maximum stress and Young’s modulus to 15.32 MPa and 151.85 MPa, respectively. This is due to the better elastomeric performance resulting from the semicrystalline aliphatic characteristics of PVL [58–61]. In addition, PVL chains can act as plasticizers and reduce the intermolecular forces between the CA chains. Compared to CA-g-PVL, the maximum strain of CA-g-PVL-Upy was slightly reduced to 97.68% and the maximum stress improved to 18.38 MPa. However, it is worth noting that Young’s modulus of CA-g-PVL-Upy was significantly improved to 331 MPa. This is due to the quadruple hydrogen bonds formed between the Upy groups.

### 3.4. Self-healing properties

**Figure 4.**
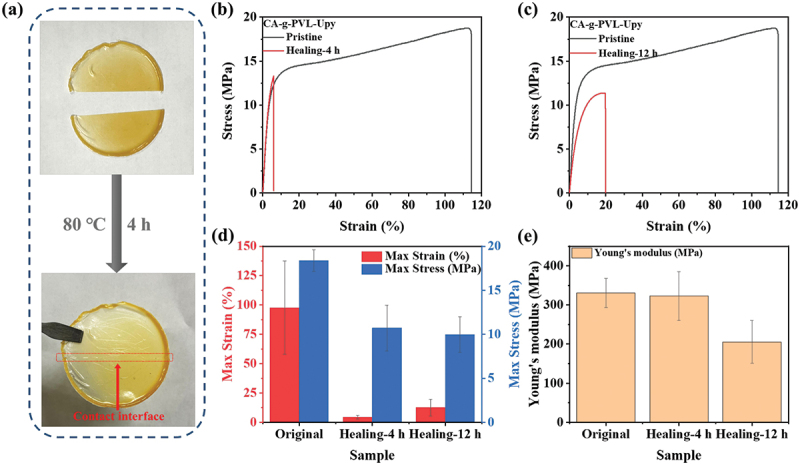
(a) Images of the self-healing process of CA-g-PVL-Upy. Strain-stress curves of CA-g-PVL-Upy healed at 80°C for (b) 4 h and (c) 12 h. (d) The maximum strain and maximum stress values obtained from the strain-stress curves. (e) Young’s moduli calculated according to the strain-stress curves.

CA-g-PVL-Upy displayed excellent self-healing performance besides improved elasticity. As shown in Figure 4(a), the two separated pieces were brought into contact at 80°C. Healing of the contact interface was achieved after 4 h by the quadruple hydrogen bonding of the Upy groups. The self-healing efficiency was evaluated by a stretching test, and the healing efficiency was calculated according to the following equation:

(4) $$\begin{array}{l} \mathrm{self}-\mathrm{healing efficiency}\\ \;=\frac{\mathrm{Max stress of the healed sample}}{\mathrm{Max stress of the original sample}} \end{array}$$

Experimental results on self-healing of CA-g-PVL-Upy are shown in Figure 4(b–e) and Figure S9. Although the maximum strain of the sample self-healed at 80°C for 4 h decreased, the maximum stress recovered to 10.71 MPa, resulting in a self-healing efficiency of 58.3%. Interestingly, extending the self-healing time to 12 h effectively improved the maximum strain of the healed sample, although the maximum stress (9.97 MPa) and self-healing efficiency (54.3%) were similar to that of the 4 h- healed sample. In addition, the sample healed for 4 h showed a high Young’s modulus of 322.85 MPa, which was close to the value of the original sample (331.00 MPa). Nevertheless, the Young’s modulus of the sample healed for 12 h decreased to 205.54 MPa. This may be due to the melting of PVL crystals during the long heating process of CA-g-PVL-Upy, which affected the toughness of the sample. CA had a *DS* of 2.4, which limited the number of hydroxyl groups for grafting PVL. Therefore, only a small amount of Upy groups can be introduced into the polymer. To rule out the influence of other compositions on the self-healing performance, CA-g-PVL was also tested. As shown in Figure S10, CA-g-PVL without Upy groups showed viscosity at 80°C due to the intense segmental motion of the PVL chains, but the separated films of CA-g-PVL were not as stable and completely repaired as CA-g-PVL-Upy. It was thus revealed that the quadruple hydrogen bonds formed between the Upy dimers played a key role in the self-healing properties. Compared to the reported self-healing polymers (Table S1), CA-g-PVL-Upy exhibited comparative mechanical performance and good self-healing efficiency despite the limited amount of Upy groups introduced into the polymer. Furthermore, it is noteworthy that CA-g-PVL-Upy was synthesized by biodegradable molecules. This is of great significance for the development of environmentally friendly self-healing materials.

### 3.4. Shape-memory properties

**Figure 5.**
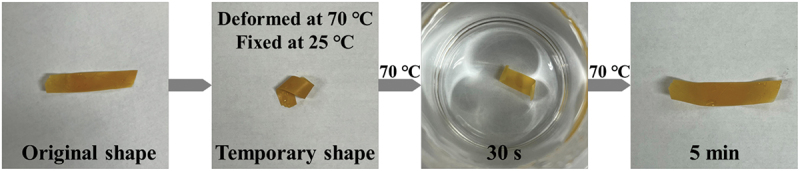
Images of shape recovery procedure of CA-g-PVL-Upy.

The CA-g-PVL-Upy also exhibited a shape-memory property. According to the DSC curve presented in Figure 2(b), CA-g-PVL-Upy showed a well-defined melting peak of crystallized PVL segments around 48°C. Therefore, the PVL segments serve as a reversible phase to store the temporal shape [62], while CA and Upy groups act as the fixed phase to maintain the original shape. In addition, 70°C could be chosen as the deformation and trigger temperature and 25°C as the fixation temperature. As presented in Figure 5, the CA-g-PVL-Upy strip was heated to 70°C and rolled into helical form as the temporal shape, which was fixed at 25°C. In particular, thermal stimulation in a 70°C water bath for 5 min quickly changed CA-g-PVL-Upy from a temporary shape to a permanent shape, which was set via the quadruple hydrogen bonding of the Upy groups and CA as the fixed phase. It was thus demonstrated that the formation of supramolecular interactions of PVL-Upy chains gives the CA-g-PVL-Upy not only excellent self-healing but also shape-memory ability.

## 4. Conclusions

In this study, a self-healing and shape memory polymer was synthesized by introducing Upy units into CA-g-PVL copolymer obtained by ring-opening graft polymerization of VL onto CA. The chemical structures, self-healing performance, and shape memory properties of the as-prepared graft polymers were comprehensively investigated. The quadruple hydrogen bonds formed between Upy groups and the semicrystalline aliphatic characteristics of PVL chains gave the resulting polymers good stretchability (97.68% of the maximum strain), self-healing (54.3% of the self-healing efficiency), and shape-memory abilities. The achievements of this study provide new insight into the synthesis of self-healing and shape-memory polymers using environmentally friendly and biodegradable materials.

## Supplementary Material

Supplemental Material
