# Supplementary material for: Self-healing and shape-memory polymers based on cellulose acetate matrix
Source: Sci Technol Adv Mater. 2024 Feb 19;25(1):2320082. doi: 10.1080/14686996.2024.2320082 (PMC10919307; doi:10.1080/14686996.2024.2320082)
Supplement: Supplemental Material [file TSTA_A_2320082_SM1952.docx]

Supporting information

Self-healing and shape-memory polymers based on cellulose acetate matrix

Han Jia^a^, Keiya Jimbo^a^, Hirogi Yokochi^b^, Hideyuki Otsuka^b^ and Tsuyoshi Michinobu^a^*

^a^Department of Materials Science and Engineering, Tokyo Institute of Technology, 2-12-1 Ookayama, Meguroku, Tokyo 152-8552, Japan; ^b^Department of Chemical Science and Engineering, Tokyo Institute of Technology, 2-12-1 Ookayama, Meguroku, Tokyo 152-8550, Japan

Correspondence email: michinobu.t.aa@m.titech.ac.jp


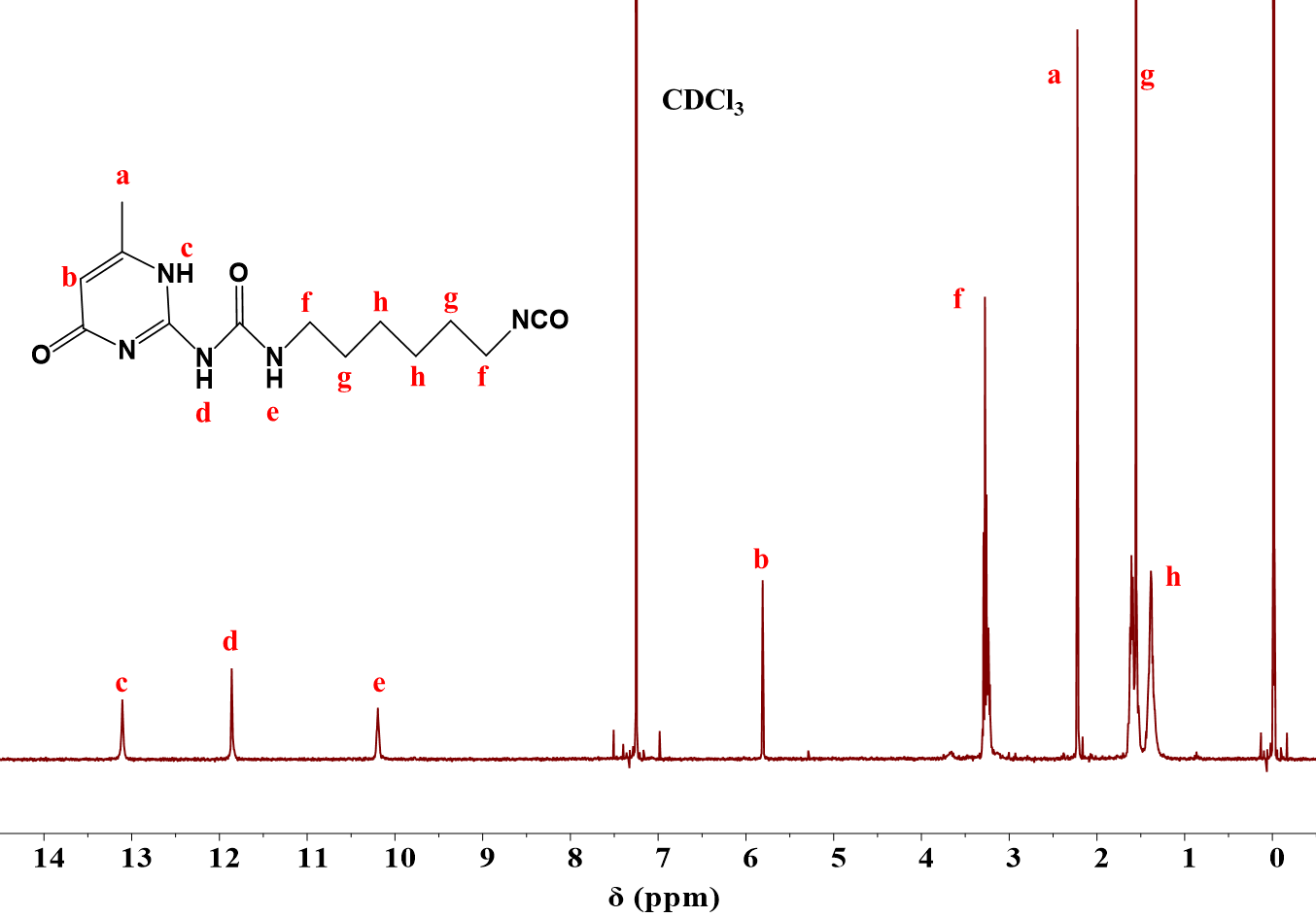


Figure S1. ^1^H NMR spectrum of Upy-NCO in CDCl_3_.


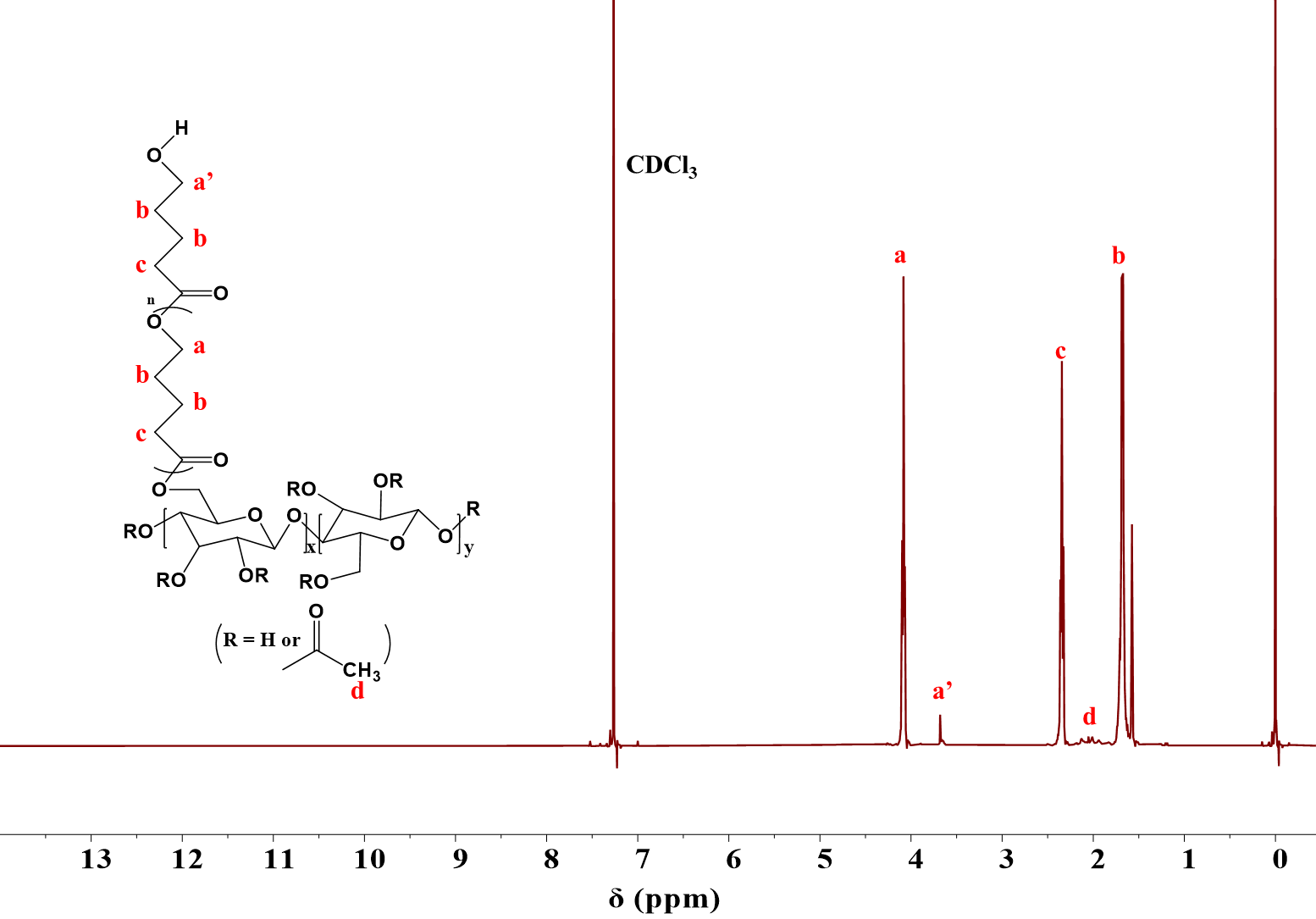


Figure S2. ^1^H NMR spectrum of CA-g-PVL in CDCl_3_.


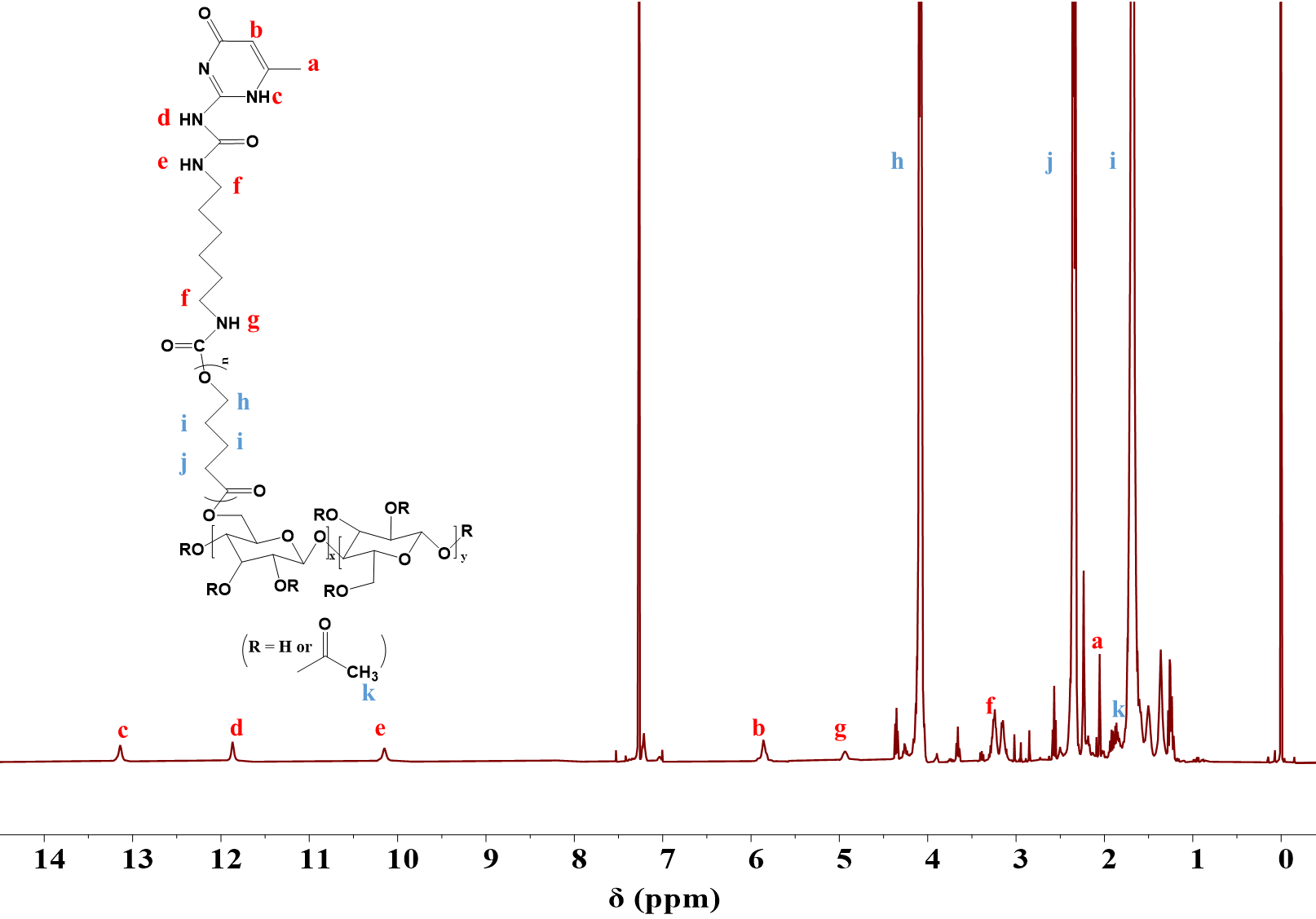


Figure S3. ^1^H NMR spectrum of CA-g-PVL-Upy in CDCl_3_.


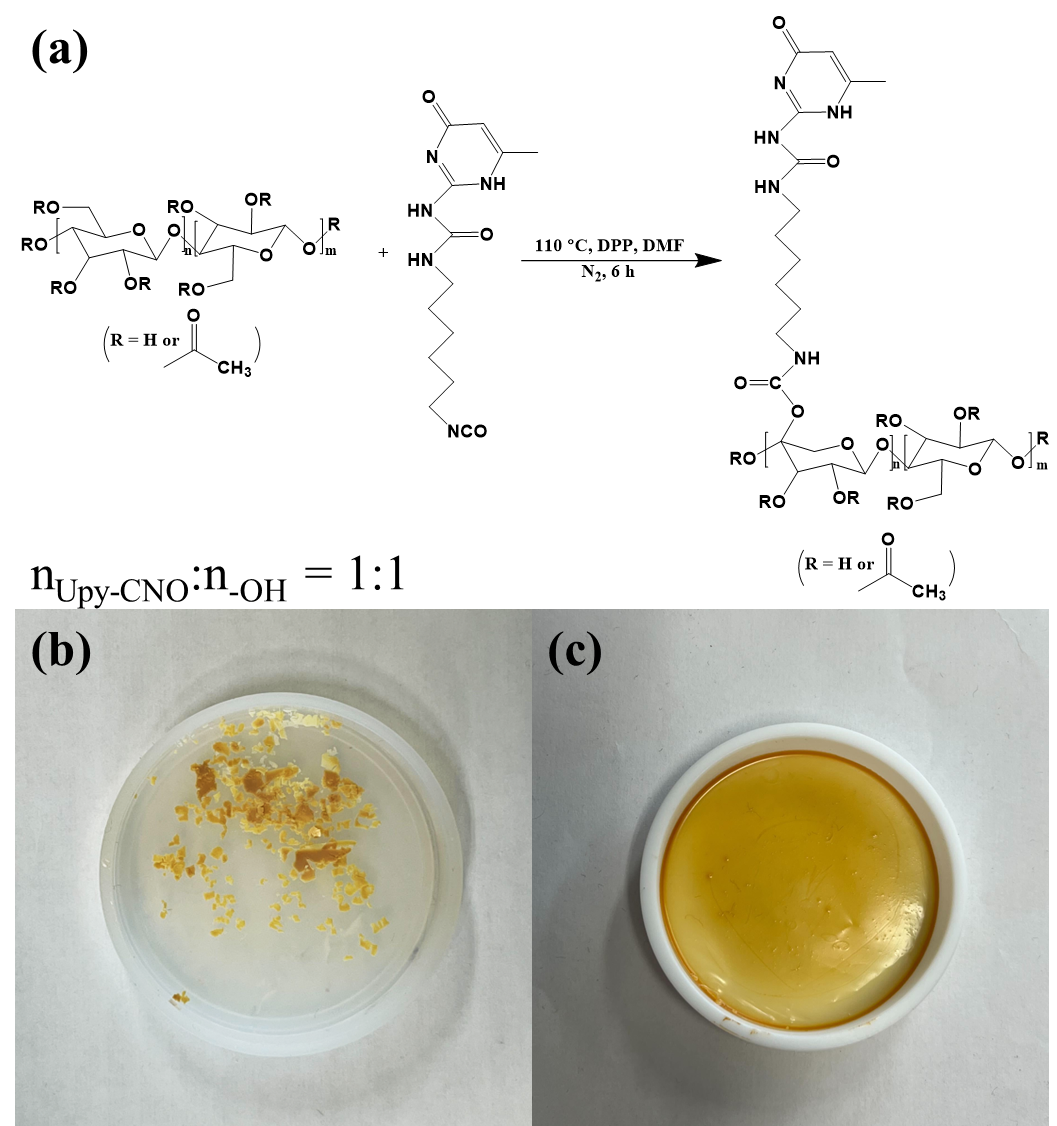


Figure S4. (a) The synthesis procedure of CA-Upy. The images of resulting products of (b) CA-Upy and (c) CA-g-PVL-Upy after removing the solvent (DMF).


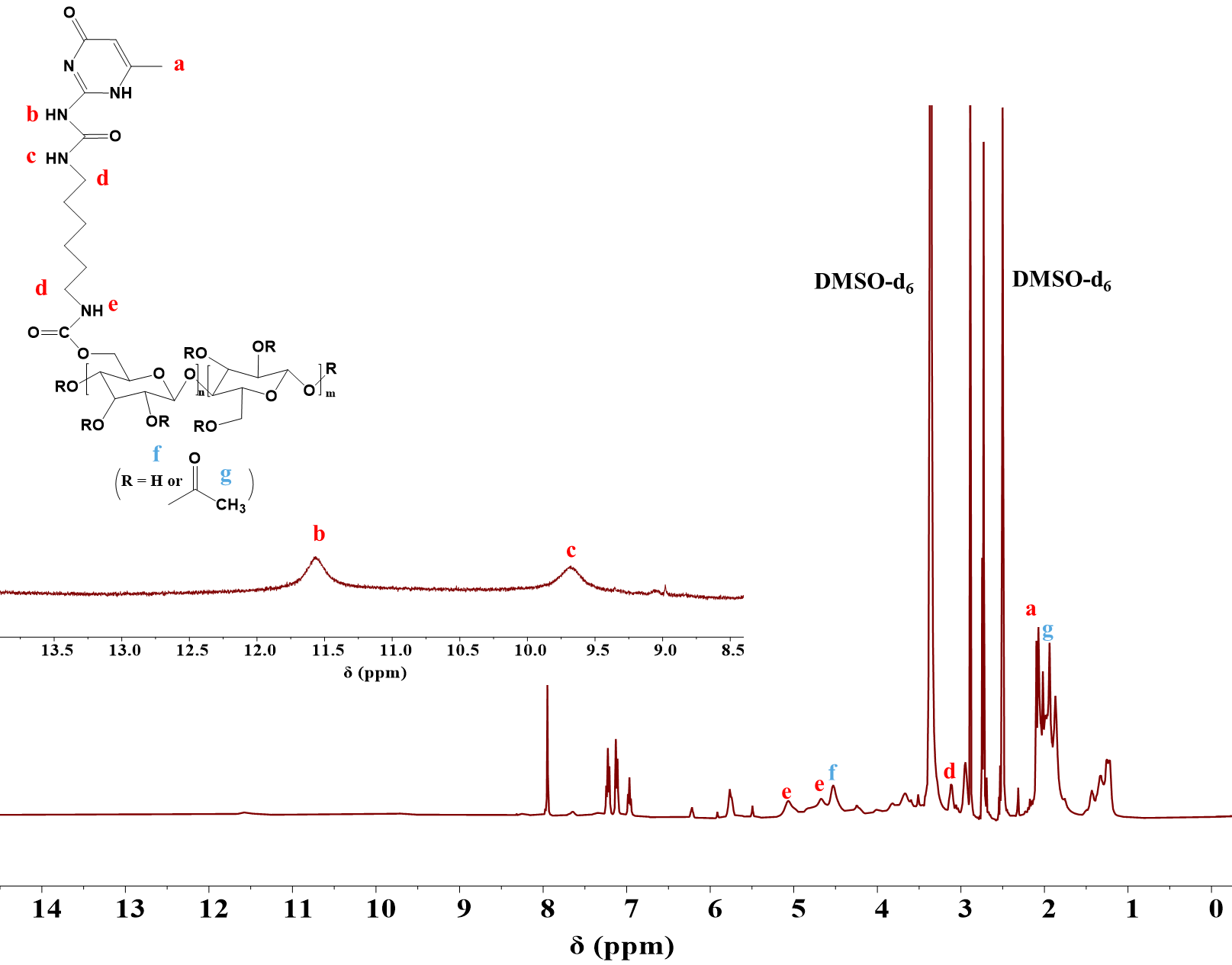


Figure S5. ^1^H NMR spectrum of CA-Upy in DMSO-d_6_.


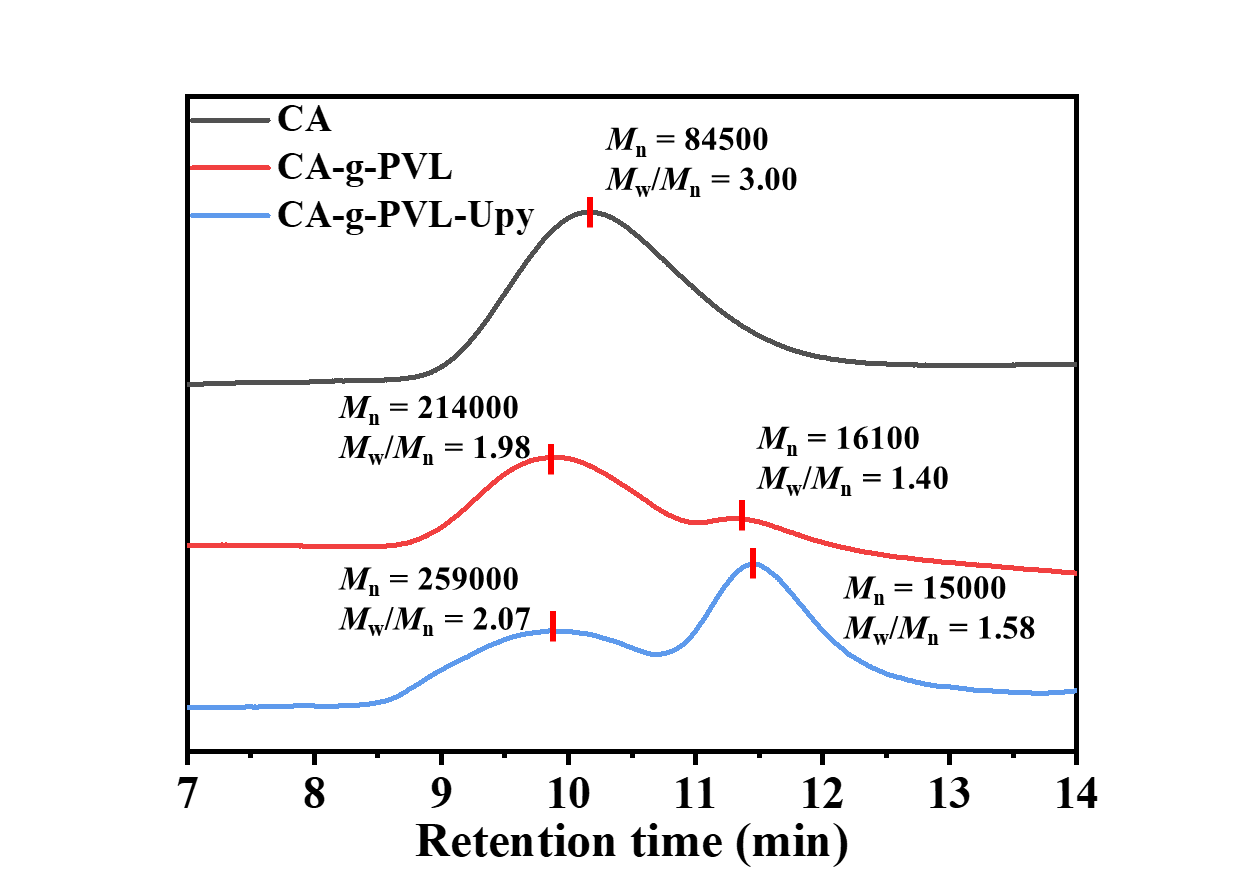


Figure S6. GPC curves of CA, CA-g-PVL, and CA-g-PVL-Upy; calibration: PS standards; eluent: DMF; flow rate: 0.6 mL/min; UV detector at 300 nm.

Figure S7. TGA curve of Upy-NCO.


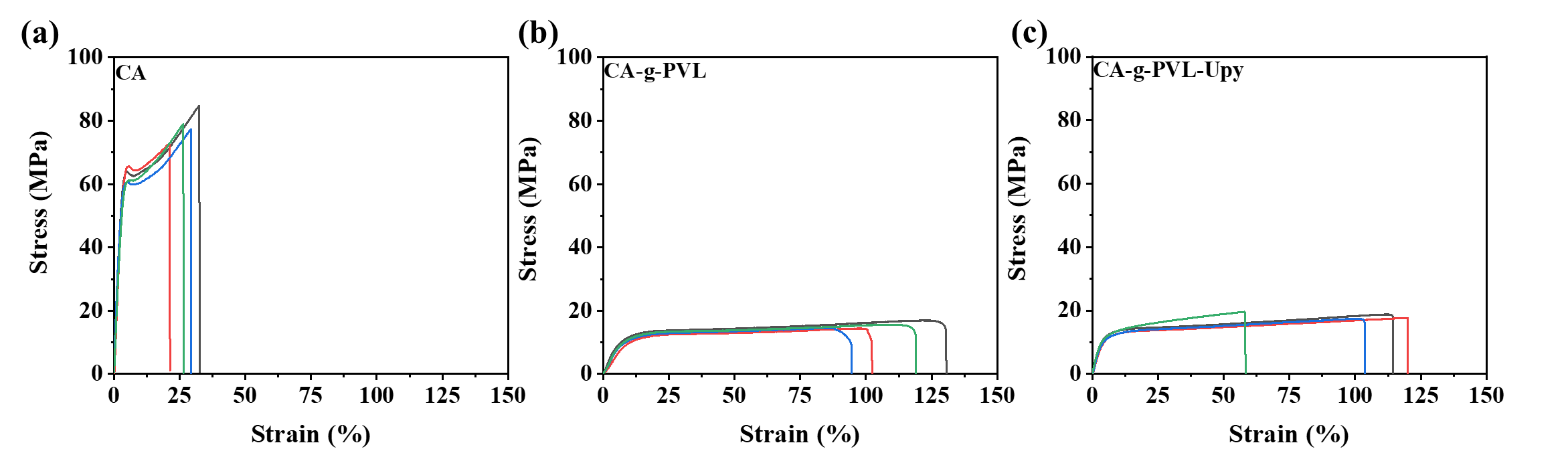


Figure S8. Strain-stress curves of (a) CA, (b) CA-g-PVL, and (c) CA-g-PVL-Upy.


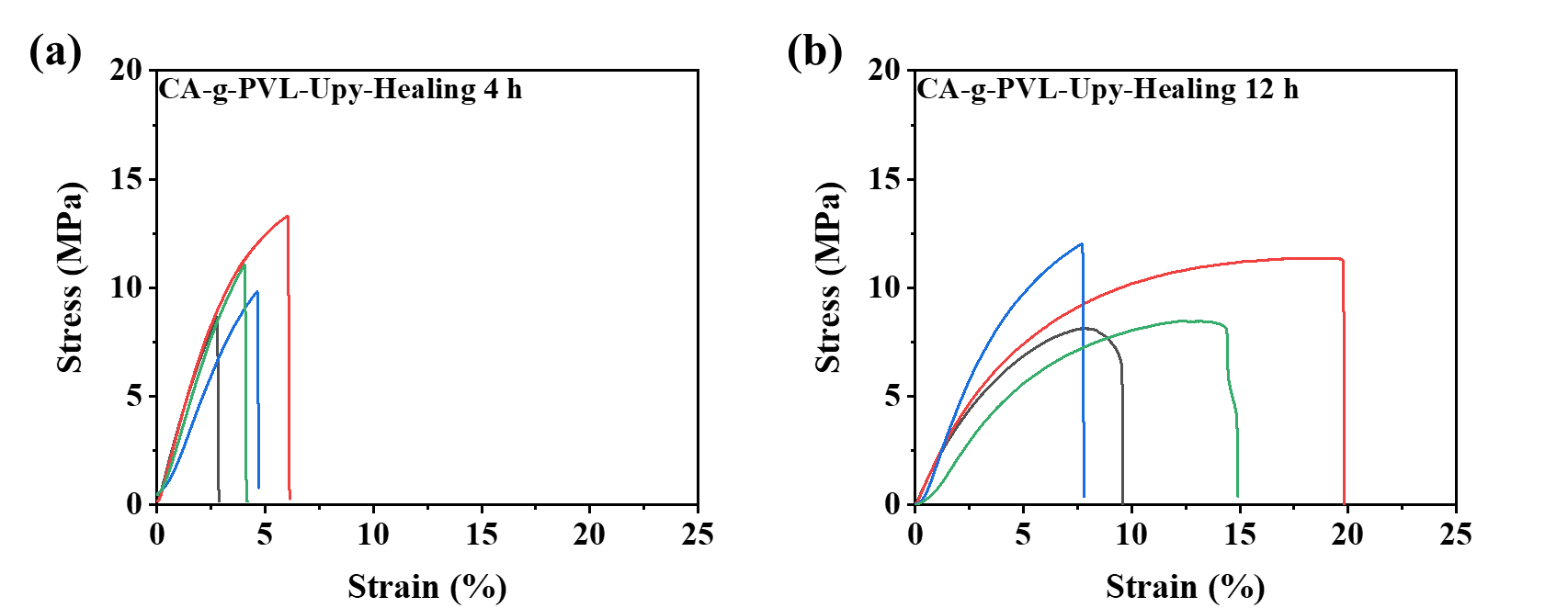


Figure S9. Strain-stress curves of CA-g-PVL-Upy after (a) 4 h and (b) 12 h of healing.


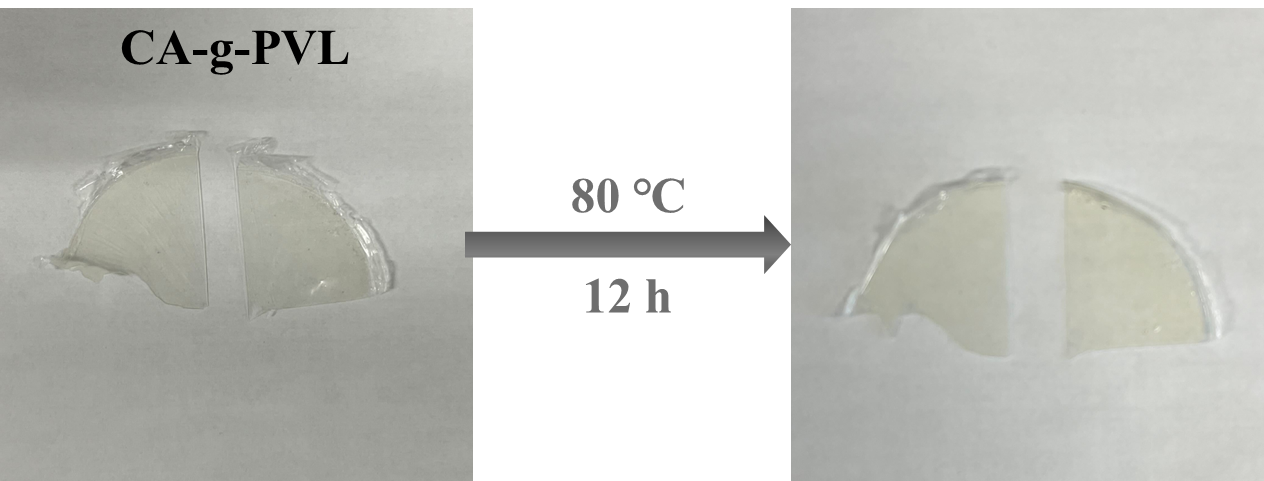


Figure S10. Images of self-healing behavior of CA-g-PVL.

Table S1. The mechanical performances and self-healing efficiencies of some reported self-healing polymers

| Sample | Healing mechanism | Pristine max stress | Pristine max strain | Self-healing efficiency | Ref. | |
| --- | --- | --- | --- | --- | --- | --- |
| NH2-MWCNTs/DA-epoxy resin | DA/r-DA reactions | 22.1 MPa | 8% | 77%  (Near-infrared irradiation) | S1 | |
|  |  |  |  | Too fragile to measure.  (Heating) |  |  |
| SSSPE | Hydrogen bonds | 2.56 MPa | 88.3% | 90.7%  (Heating) | S2 | |
| Diselenide samples | Diselenide bonds | 2.0 MPa | 700% | 76%  (Room temperature) | S3 | |
| Disulfide samples | Disulfide bonds | 0.86 MPa | 560% | 43%  (Room temperature) | S3 |  |

**References**

[S1] Li QT, Jiang MJ, Wu G, et al. Photothermal conversion triggered precisely targeted healing of epoxy resin based on thermoreversible Diels-Alder network and amino-functionalized carbon nanotubes. ACS Appl. Mater. Interfaces 2017;9:20797−20807. doi: 10.1021/acsami.7b01954

[S2] Jo YH, Zhou B, Jiang K, et al. Self-healing and shape-memory solid polymer electrolytes with high mechanical strength facilitated by a poly(vinyl alcohol) matrix. Polym. Chem. 2019;10:6561−6569. doi: 10.1039/c9py01406c

[S3] An X, Aguirresarobe RH, Irusta L, et al. Aromatic diselenide crosslinkers to enhance the reprocessability and self-healing of polyurethane thermosets. Polym. Chem. 2017;8:3641-3646. doi: 10.1039/C7PY00448F
